# Imaging findings of atypical leiomyoma of urinary bladder simulating ureterocele

**DOI:** 10.1093/jscr/rjac256

**Published:** 2022-06-22

**Authors:** Nojoud AlAmri, Hussam Abdullah, Fawaz AlIbrahim, Khadijah Eid, Mohammed AlShehri

**Affiliations:** Department of Urology, King Abdullah bin Abdulaziz University Hospital, Princess Nourah Bint Abdulrahman University, Riyadh 11552, Saudi Arabia; Department of Urology, King Abdullah bin Abdulaziz University Hospital, Princess Nourah Bint Abdulrahman University, Riyadh 11552, Saudi Arabia; Department of Urology, King Abdullah bin Abdulaziz University Hospital, Princess Nourah Bint Abdulrahman University, Riyadh 11552, Saudi Arabia; Department of Urology, King Abdullah bin Abdulaziz University Hospital, Princess Nourah Bint Abdulrahman University, Riyadh 11552, Saudi Arabia; Department of Urology, King Abdullah bin Abdulaziz University Hospital, Princess Nourah Bint Abdulrahman University, Riyadh 11552, Saudi Arabia

## Abstract

Bladder leiomyomas are relatively rare benign tumors of the bladder. They represent <0.5% of all urinary bladder tumor. In the literature, only about 250 cases reported worldwide. They are commonly found in middle-aged females. Patients present with variety of clinical presentations with predominance of obstructive urinary symptoms. Ultrasound bladder is the first preferable diagnostic tool. Definitive diagnosis is made by histopathological examination. Surgical excision is almost highly effective, leaving a low recurrence rate. We report a 34-year-old male who presented with complaints of dysuria and incomplete voiding. Ultrasound showed a cystic fluid-filled bladder mass (ureterocele), which was excised through a transurethral resection. The histopathologic diagnosis was bladder leiomyoma.

## INTRODUCTION

Mesenchymal bladder tumors represent 1–5% of all urinary bladder neoplasms. Leiomyomas encompass 35% of this subset and 0.43% of all bladder tumors [1]. It was first described by Kretschmer et al. in 1931 [2]. The prevalence of these tumors is higher in women than men with a ratio of 2:1 [2]. They affect patients in their fourth to fifth decades of life. The etiology of leiomyomas is still unknown. Leiomyomas may be categorized as endovesical, extravesical or intramural. Endovesical masses have been mostly recognizable (63%); possibly due to its characteristic bulging into the bladder which induced irritative symptoms and forced the patient to seek medical treatment, whereas intramural leiomyomas are present in 3–7% and extravesical in 11–30% [2]. Bladder leiomyomas are symptomatic in 80% of patients. Symptoms range from obstructive urinary symptoms (49%), irritative urinary symptoms (38%), hematuria (11%) and rarely it can cause flank pain and dyspareunia. US bladder is used as the primary imaging modality; however, computed tomography (CT) and magnetic resonance imaging (MRI) may be useful in determining location or the tumor, depth of invasion, size as well as assessing areas suspicious for malignancy. MRI by itself could confirm this diagnosis, but it cannot differentiate mesenchymal tumors from the more common transitional cell tumors, and the histopathological study is always necessary to confirm the diagnosis. Surgical excision has excellent prognosis after complete resection.

## CASE REPORT

**Figure 1:**
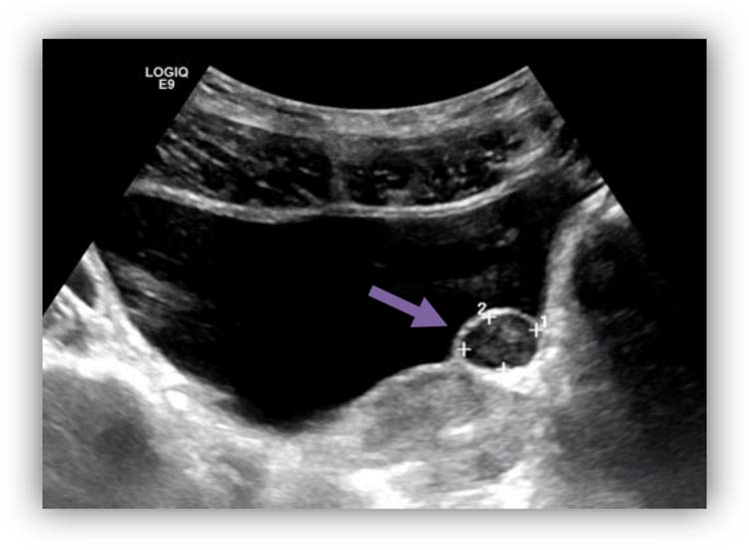
US bladder showed a cystic fluid-field bladder mass of 15 mm × 11 mm over the area of left vesicoureteric junction suggestive of left ureterocele.

In a university hospital, we recently treated a 34-year-old gentleman who presented to urology clinic complaining of repeated episodes of dysuria and incomplete voiding for the last 3 months. During the initial evaluation, no abnormalities were found on physical examination or in routine laboratory studies. Ultrasound of the abdomen and pelvis (Fig. 1) showed a hyperechogenic cystic fluid-filled mass over the area of left vesicoureteric junction measures of 15 mm × 11 mm with no intraluminal lesions or debris. These findings are suggestive for left ureterocele. Pre-void volume of 257 ml, post-void volume of 22 ml and prostate size of 17.7 ml. No hydroureteronephrosis.

**Figure 2:**
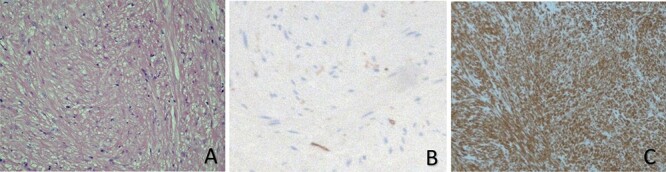
Histopathological studies show that there is a proliferation of spindle-shaped cells, in addition to an eosinophilic cytoplasm and fibers, with no evidence of mitotic changes or atypia (**A**). Leiomyomas of the urinary bladder also stain negatively for Ki-67 (**B**), but they are positive for smooth muscle staining (actin) (**C**).

The patient underwent cystoscopy, which revealed a lesion protruding into the bladder lumen from the left lateral and anterior wall of the urinary bladder with normal covering urothelium. The patient underwent an uneventful transurethral resection of the bladder tumor (TURBT). Both ureteric orifices were seemed to be intact. The lesion was solid in consistency with no fluid content. The catheter was removed on the fifth postoperative day and the patient was discharged without any problem. Pathology proved that the lesion was a benign submucosal leiomyoma (Fig. 2A–C).

Patient was seen at the urology outpatient clinic2-week postoperative. The patient experienced a relief of symptoms and normal voiding function was retained after surgical excision. He was given appointment in 3 months with new ultrasound. Patient remains asymptomatic up to the time of writing this case report.

## DISCUSSION

Bladder benign mesenchymal tumors are relatively rare representing (1–5%) of all urinary bladder neoplasms [3]. Leiomyomas are benign smooth muscle tumors that can occur in any organ [4]. Leiomyomas of the urinary bladder account for <0.43% of all bladder tumors, with only 250 cases have been reported in the English literatures [2, 4]. Despite the increased availability of high-resolution imaging modalities, bladder leiomyomas remain rare tumors [5]. The incidence of bladder leiomyoma in women is twice as high as that in men [2]. They occur mainly between the fourth and fifth decades of life [6]. However, there are cases described in younger patients. The etiology of these benign tumors is still unknown, and multiple theories such as hormonal, chromosomal alteration and chronic inflammation try to explain their origin [5]. They remain asymptomatic in 20% of patients. Majority of cases present with obstructive urinary symptoms (49%), irritative urinary symptoms (38%), hematuria (11%) and rarely it can cause dyspareunia [4, 5]. They might cause flank pain and hydronephrosis on imaging as tumor obscuring the ureteric orifice [4]. These tumors are classified into three different locations, i.e. endovesical, intramural and extravesical. Most of the symptomatic patients tend to have endovesical tumors (63–86%), whereas intramural leiomyomas are present in 3–7% and extravesical in 11–30% [4, 5]. Endovesical tumors are usually polypoid or pedunculated, whereas intramural bladder leiomyomas are well encapsulated lesions and surrounded by the bladder wall muscle. The endovesical form will cause irritative or obstructive symptoms, and gross hematuria, resulting in a relatively early diagnosis [5]. Bladder ultrasound considered the most useful and the first choice as imaging diagnostic tool. In most cases, it shows a small, solid and encapsulated mass with homogeneous and hypoechoicity [4]. CT and MRI are used to delineate the location, the size, extent of the tumor and their relation adjacent structures. Despite the imaging characteristic features, the definitive diagnosis is by histopathology. Pathological diagnosis is important to differentiate leiomyomas from other bladder pathology such as leiomyosarcoma or inflammatory myofibroblastic tumor [1, 4]. Bladder leiomyomas could be easily misdiagnosed radiologically with many other urinary bladder tumors, cystocele, ureterocele, uterine fibroids and other pelvic masses [7]. The treatment of bladder leiomyoma is surgical excision. The approach of excision depends on the site and size of the tumor. In small and easily accessible tumors, the transurethral resection of bladder tumor is preferred in 90% of cases. In large tumors that invade the bladder wall, segmental resection or laparoscopic partial cystectomy might be necessarily [5]. Trans-vaginal route has been reported in the literatures for extravesical tumors adjacent to the vagina [7]. Overall, 18% of patients in the TURBT group required a reoperation due to incomplete resection of the tumor [1]. It is mandatory to make sure that the ureteric orifice will not be obstructed by any residual tumor, retrograde pyelogram and inserting a ureteric stent is advisable to avoid the postoperative local edema causing a persistent pelvicalyceal dilatation [5]. Overall prognosis is good with almost no recurrence when fully resected [5]. There are no guidelines regarding the follow-up of these tumors, and the clear suggestion is to avoid exposing the patients to invasive or radiologic investigations to detect small asymptomatic recurrences postoperatively. Histopathologically, leiomyomas of the urinary bladder are composed of fascicles of smooth muscle fibers that are separated by connective tissues. They are non-infiltrative smooth muscle benign tumors with no mitotic activity, cellular atypia or necrosis. On immuno-histochemistry, they will have positive staining for smooth muscle actin and negative staining for Ki-67 [5].

## CONCLUSION

In summary, this case study describes an unusual imaging finding of bladder leiomyoma. It is very rare and difficult to be identified on US, CT, MRI and cystoscopy. A definitive diagnosis of the bladder lesion depends on biopsy and histopathological study. However, it is necessary to take into account the possibility that the bladder cystic lesions may have differential diagnosis of bladder leiomyoma.

## CONFLICT OF INTEREST STATEMENT

None declared.

## FUNDING

None.
